# COVID-19 Neurological Manifestations and Underlying Mechanisms: A Scoping Review

**DOI:** 10.3389/fpsyt.2020.00860

**Published:** 2020-08-21

**Authors:** Angela Wenting, Angélique Gruters, Yindee van Os, Sonja Verstraeten, Susanne Valentijn, Rudolf Ponds, Marjolein de Vugt

**Affiliations:** ^1^Department of Medical Psychology, Catharina Hospital, Eindhoven, Netherlands; ^2^Department of Psychiatry and Neuropsychology, School for Mental Health and Neuroscience, Alzheimer Center Limburg, Maastricht University, Maastricht, Netherlands; ^3^Department of Medical Psychology, Elkerliek Hospital, Helmond, Netherlands; ^4^Department of Medical Psychology, Máxima Medical Center, Veldhoven, Netherlands; ^5^Department of Psychiatry and Neuropsychology, School for Mental Health and Neuroscience, Maastricht University Medical Center, Maastricht, Netherlands

**Keywords:** Corona Virus Disease 2019, neurological, neurotropic, cognitive, scoping review

## Abstract

**Background:**

In the Corona Virus Disease 2019 (COVID-19) pandemic, the primary problem is respiratory-related, but there also is increasing evidence of central nervous system (CNS) involvement. This study aims to summarize the literature on neurological manifestations of COVID-19, underlying mechanisms of CNS involvement and cognitive consequences.

**Methods:**

A scoping review was conducted with multiple searches in PubMed, PsycInfo, and CINAHL databases. Full text articles in English were included if they involved humans with COVID-19. The search was updated twice, the latest on 19 May 2020.

**Results:**

After screening 266 records and cross referencing, 85 articles were included. The articles were case studies, opinion papers, letters to editors, and a few observational studies. No articles were found regarding cognitive consequences in COVID-19 patients. All reported on neurological manifestations and/or underlying mechanisms of CNS involvement in COVID-19.

**Conclusion:**

Neurological manifestations of COVID-19 vary from mild (e.g. loss of taste and smell, dizziness, headache) to severe (e.g. ischemic stroke, encephalitis). Underlying pathways are suggested to be both indirect (as a result of thrombotic complication, inflammatory consequences, hypoxia, blood pressure dysregulation), and direct (neurotropic properties of the virus). Since most articles were opinion papers and no studies have been conducted on cognitive consequences, further research is warranted.

## Introduction

The recent outbreak of Corona Virus Disease 2019 (COVID-19) caused by the Severe Acute Respiratory Syndrome Corona Virus 2 (SARS-CoV-2) led to the current pandemic, which is characterized by ominously high infection rates. By the end of May, over 4.8 million people have been infected and over 323,000 deceased worldwide due to COVID-19 (1). To stop the devastating impact of COVID-19, scientists are in a race to find a cure or vaccine for the virus.

SARS-CoV-2 is primarily transmitted between people through respiratory droplets. It can bind to the angiotensin-converting enzyme 2 (ACE2) receptor in the lungs. After which most people develop mild symptoms, such as coughing or fever. However, the disease can lead to more severe problems such as pneumonia (2). In the Netherlands, a quarter of all identified patients were admitted to the hospital due to COVID-19. The leading cause of hospital admittance was respiratory failure due to acute respiratory distress syndrome (ARDS) (3, 4).

Although the most prominent symptoms of COVID-19 are respiratory-related, there is also emerging literature on neurological manifestations of the virus. First opinion papers, letters to the editors, and case studies have been published. The primary aim of the current study was to summarize the literature on neurological manifestations due to COVID-19 and its underlying mechanisms. Furthermore, if COVID-19 might lead to neurological tissue damage, then it could lead to impaired cognitive functioning (e.g. memory impairment or attention problems). Therefore, the secondary aim was to summarize the literature on cognitive consequences of COVID-19.

## Methods

### Design

A scoping review was used to provide an overview of the relevant literature on neurological and cognitive manifestations in COVID-19 patients (5). Possible mechanisms underlying these manifestations will be presented based on the available evidence. A scoping review was chosen over a systematic review to provide a broader overview of the literature using multiple sources (e.g., opinion papers, letters to the editors, case studies). We used the extended PRISMA checklist for scoping reviews and the following methodological framework: identifying the research question, study selection, charting the data, and reporting the results (5–7).

### Data Sources, Search Strategy, and Inclusion and Exclusion Criteria

The databases PubMed, PsycInfo, and CINAHL were searched using a search strategy based on free text terms in Title/Abstract and descriptor terms. Some basic searches were carried out by two authors to identify key terms. The search strategy is provided in the **Supplementary Material**. We included all relevant publications on the neurological manifestations in COVID-19, underlying mechanisms, and cognitive consequences. A limit was placed on year of publication (2019–2020). Animal studies, studies in neonates, and articles without a full text in English were excluded. The search was carried out on 29 April 2020 and updated on 12 May and 19 May 2020.

### Study Selection and Charting of the Data

One author independently screened all titles and abstracts. Two authors discussed the included full texts and found a perfect agreement on the included papers. Cross referencing was applied to determine if relevant articles were missing. Two authors reviewed, extracted, and summarized the full text articles. The main topics were analyzed using a qualitative content approach and narratively described. The following themes were determined: neurological manifestations in COVID-19 and its underlying mechanisms. Descriptives were reported from the observational studies.

## Results

**Figure 1:**
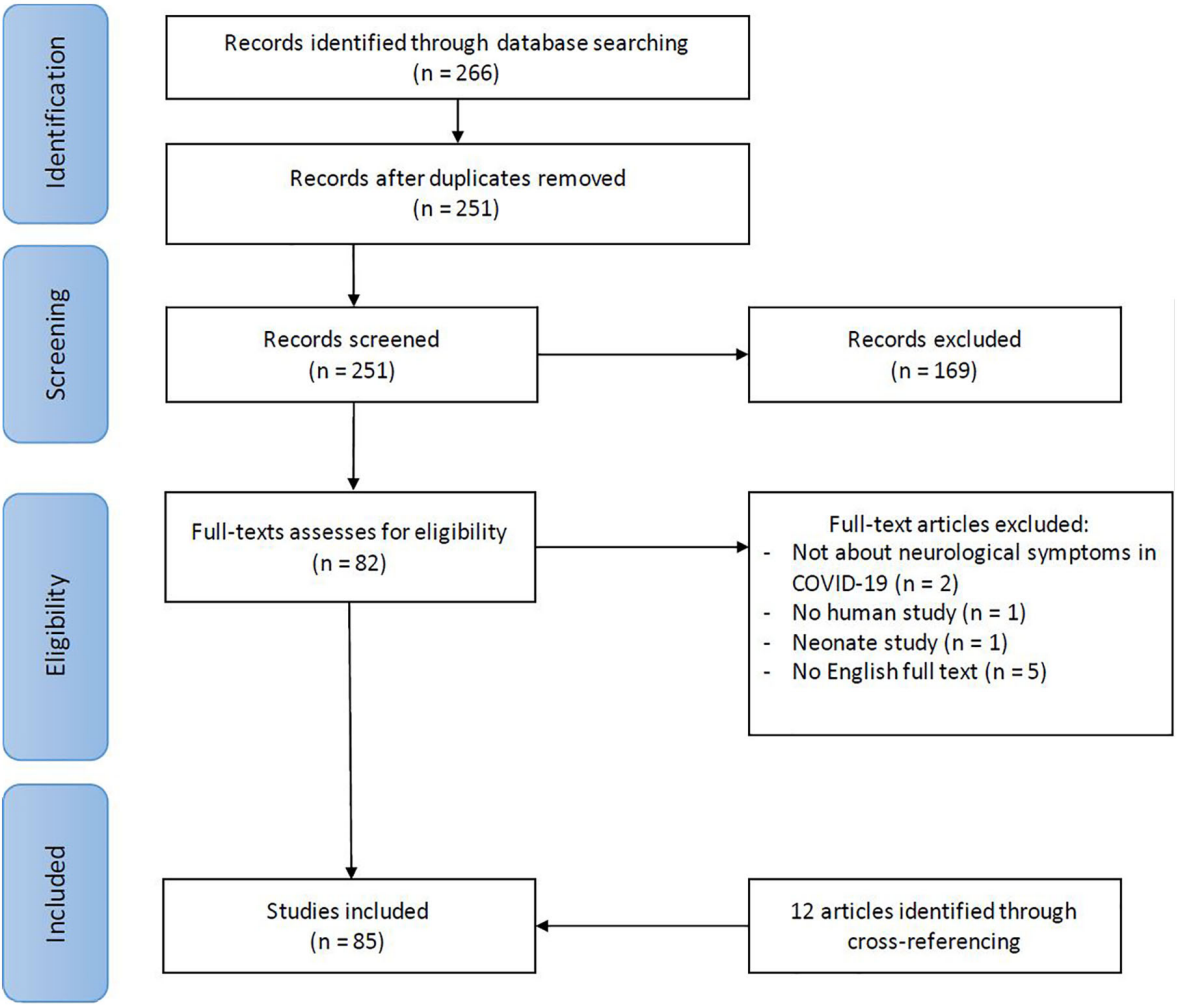
Flowchart article selection procedure.

The first search (29 April) yielded 160 records, the second update (12 May) 206 records, and the final update (19 May) 266 records. A total of 82 full texts were evaluated for inclusion, of which 73 were included. Through cross referencing another twelve articles were identified, which led to the inclusion of 85 papers. **Figure 1** shows the flowchart of this selection process. In this scoping review, all included papers were summarized using a narrative report. An overview of the findings of all articles are described in **Table 1** in the **Supplementary Material**. The 85 articles were 25 opinion papers, 22 case studies, 16 letters to the editor, 13 reviews (11 literature reviews, 1 systematic review, 1 scoping review), 6 observational studies, and 3 comments on other publications.

### Neurological Manifestations and Neuropsychological Consequences

Unfortunately, no papers have been reported yet on cognitive consequences of COVID-19, such as memory impairment or attention deficits in COVID-19 patients. However, the literature on neurological manifestations of SARS-CoV-2 is emerging. A few observational studies on neurological symptoms have been conducted in COVID-19 patients. Mao, Wang (8) demonstrate that 36.4% of 214 hospitalized COVID-19 patients had neurological symptoms, varying from dizziness and headache, to cerebrovascular disease. In the COVID-19 intensive care (IC) population neurological symptoms were found in 84 percent of 58 patients included in the study of Helms et al. (9), and 21 percent of 235 patients included in the study of Kandemirli, Dogan (10).

**Figure 2:**
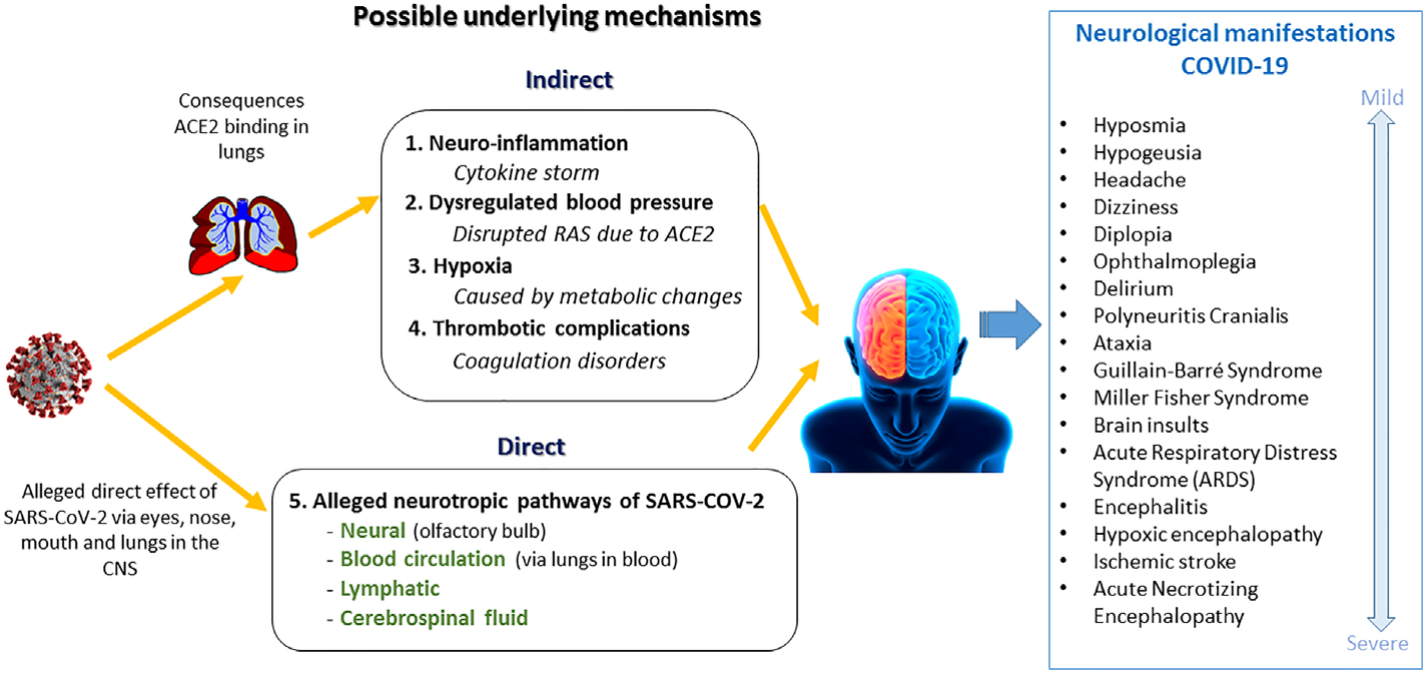
Neurological manifestations of COVID-19 and possible underlying mechanisms.

There is a great variety in the type and severity of the neurological manifestations of COVID-19. The first evidence of mild neurological symptoms emerged quickly after the outbreak, such as hyposmia, hypogeusia, headache, dizziness, diplopia, and ophthalmoplegia (11–18). Case studies have been published since February on neurological symptoms as an atypical presentation of COVID-19 (19). To illustrate, a delirium could be a first atypical symptom of COVID-19, especially in the elderly (20–22). Neurological movement disorders have also been reported in COVID-19 cases, such as Guillain-Barré syndrome, Miller Fisher syndrome, polyneuritis cranialis, and ataxia (8, 23, 24). Furthermore, Lu, Xiong (25) found acute symptomatic seizures in 27% of 304 COVID-19 patients with no prior history of seizures. More severe types of brain disease (encephalopathy) have been described as well, such as hypoxic encephalopathy, encephalitis, and stroke (11–13, 26). A retrospective study from Chen, Wu (27) showed that 20% of 113 COVID-19 ICU patients had hypoxic encephalopathy. In another study 31% of 184 ICU patients with COVID-19 had thrombotic complications with ischemic strokes (28). Furthermore, acute necrotizing encephalopathy (ANE) is also described in a case report of a COVID-19 patient (29). ANE is a rare disease, which can lead to severe brain damage including hemorrhage. In conclusion, neurological manifestations vary from mild to severe, all summarized in **Figure 2**.

### COVID-19 Mechanisms Underlying the Neurological Manifestations

The literature about possible pathways in which COVID-19 can cause neurological manifestations is emerging. Both direct and indirect suggested pathways are summarized in **Figure 2**.

The suggested indirect effects of SARS-CoV-2 on the brain are:

1. Neuro-inflammation: a cytokine storm, induced by the immune system in reaction to the virus, can spread through the body, pass the blood brain barrier, and can cause brain infections or damage nearby neurons and glial cells (20, 30–38).
2. Blood pressure imbalance: since ACE2 regulates blood pressure in the renin angiotensin aldosterone system (RAS), damage of the ACE2 receptor can lead to hypertension or hypotension (30, 38–41).
3. Hypoxia: metabolic disruption, caused by lung damage, can lead to an oxygen deficiency in the brain (30, 32, 37, 38).
4. Thrombosis: thrombotic complications can lead to ischemic strokes (32, 38).

First opinion papers are published in which scientists assume that SARS-CoV-2 can enter the central nervous system CNS (12, 14, 16, 17, 27, 42, 43). It is stated that other coronaviruses have been found to be neurotropic (16, 27, 42, 44). Hereby ACE2, to which the virus is binding to, is assumed also to be present in the brain (42–44). In a case study in two patients, SARS-CoV-2 was not found in the cerebrospinal fluid (CSF) (45). However, two other case studies mentioned presence of the virus in the frontal lobe and CSF (39, 40). Various direct pathways are proposed to which the virus could possibly have a direct effect on the brain. After the virus enters the body *via* the eyes, nose, or mouth, it can bind to ACE2 receptors. The possibility of binding to the ACE2 receptors in the nose and taste buds could explain the possible loss of taste and smell in COVID-19 (16). A neural pathway is suggested, in which the virus enters the CNS along the olfactory pathway penetrating the olfactory bulb in the forebrain (15, 30–32, 34, 35, 46–48). Also, the virus could be transported to the brain *via* the lymphatic system through lymphoid tissue (32, 43, 49). The blood circulation is another supposed pathway. When the virus enters the blood circulation it can be transported to the brain. The virus could possibly pass the blood brain barrier by infecting the endothelial cells (49). Once in the CNS, the virus could possibly enter the cerebrospinal fluid, through which it can spread through the brain (15, 30, 35). The medulla oblongata is the primary respiratory control center, located in the lowest portion of the brain stem. It is suggested that damage to the medulla oblongata, caused by direct effects of SARS-CoV-2, can possibly explain respiratory failure in COVID-19 (20, 32, 37, 43, 46, 48, 50–53). Scientists propose that this neurotropic potential of SARS-CoV-2 may account for the difference in respiratory distress in patients (51). However, some researchers believe this is not the case, since recovery of respiratory distress symptoms takes place (32).

## Discussion

The aim of the current study was to summarize the literature on neurological manifestations in COVID-19, underlying mechanisms, and cognitive consequences. This scoping review included 85 articles. Neurological manifestations were prominently described with suggestions for underlying mechanisms. The CNS is involved in COVID-19, as neurological manifestations (e.g., consequences of hypoxia and thrombosis) were shown in several case reports and observational studies (7, 8, 25, 53, 54). Suggested pathways of CNS involvement are both direct (neurotropic) and indirect. Since no literature on cognitive consequences of COVID-19 was found yet, previous research on other coronaviruses should be taken into account. A systematic review and meta-analysis on recovered patients of other coronaviruses found that a significant proportion of patients developed a delirium during the acute stage, and almost halve (44%) had a memory impairment post-illness (54). Elderly, people with already existing neurodegenerative diseases, or people with psychiatric comorbidities might be even more at risk for cognitive impairment following COVID-19, due to their cognitive vulnerability. Hereby, since people living with dementia might have difficulties in understanding and remembering the public health information, they are more vulnerable to be infected with COVID-19 (55, 56). In case of co-occurrence of COVID-19 and dementia, delirium could complicate the presentation of dementia (55, 56). Furthermore, as a result of neuro-inflammation that causes or progresses neurodegenerative processes in the brain, it is suggested that COVID-19 could result in a higher incidence of neurodegenerative diseases (30).

A strength of the current study is the use of scoping review methodology to gain insight into the current available evidence on neurological manifestations and underlying mechanisms while using a systematic process with a replicable and transparent approach. To our knowledge, this is the first scoping review on both neurological manifestations, its underlying mechanisms, and cognitive consequences in COVID-19 patients. Some limitations have to be considered as well. No quality appraisal of the studies was taken into account, which is often the case in scoping reviews. Due to the quick rise of literature on COVID-19 new publications might have emerged. The body of literature is fast-growing, which is illustrated by the inclusion of 27 articles after our first search on the 29 April 2020, and the latest update on 19 May 2020 resulting in a total of 85 articles.

## Therapeutic Implications

- Clinicians should be vigilant for CNS involvement and possible neurological manifestations of COVID-19.
- Clinicians should be aware of possible neurological and cognitive complaints post-COVID-19, especially in older patients, patients with cognitive impairment and/or psychiatric comorbidity.
- In case of cognitive complaints post-COVID-19 cognitive screening or neuropsychological assessment is recommended.
- In case of complex cognitive of emotional complaints post-COVID-19 it is recommended to involve a psychiatrist or psychologist.

## Conclusion and Recommendations

In conclusion, neurological manifestations of COVID-19 vary from mild, such as headache and dizziness, to severe, such as ischemic stroke and encephalitis. Underlying mechanisms of CNS involvement are suggested to be both direct (neurotropic) and indirect (as a result of thrombotic complication, inflammatory consequences, hypoxia, blood pressure dysregulation). Since most articles were opinion papers, further research is warranted.

No literature was found on the cognitive consequences of COVID-19. Therefore, cross sectional and longitudinal studies are needed. Neuropsychological assessment could be used to monitor the course of cognitive functioning after recovering from COVID-19. This should not only be conducted in hospitalized COVID-19 patients, but should also contain community-based studies in adults and children that recovered from COVID-19.

## Author Contributions

AW and AG carried out abstract screening and full text extraction of the scoping review. The first draft of the manuscript was written by both AW and AG. All other authors contributed to revisions of the manuscript.

## Conflict of Interest

The authors declare that the research was conducted in the absence of any commercial or financial relationships that could be construed as a potential conflict of interest.
