# Supplementary material for: COVID-19 Neurological Manifestations and Underlying Mechanisms: A Scoping Review
Source: Front Psychiatry. 2020 Aug 21;11:860. doi: 10.3389/fpsyt.2020.00860 (PMC7472775; doi:10.3389/fpsyt.2020.00860)
Supplement: Supplementary file 1 [file Table_1.docx]

Supplementary Material

**Search strategy**

*PubMed*

('covid-19'[Title/Abstract] OR 'covid 19'[Title/Abstract] OR ‘2019-ncov’[Title/Abstract] OR 'severe acute respiratory syndrome coronavirus 2'[Title/Abstract] OR 'severe acute respiratory syndrome cov 2'[Title/Abstract] OR 'sars coronavirus 2'[Title/Abstract] OR 'sars cov 2'[Title/Abstract] OR ‘coronavirus’[Title/Abstract] OR "COVID-19" [Supplementary Concept]) AND ('neuropsychological'[Title/Abstract] OR 'neuropsychology'[Title/Abstract] OR 'cognitive'[Title/Abstract] OR 'cognition'[Title/Abstract] OR 'brain'[Title/Abstract] OR 'neurological'[Title/Abstract] OR ‘encephalitis’)

*PsycInfo/CINAHL*

AB ('covid-19' OR 'covid 19' OR ‘2019-ncov’OR 'severe acute respiratory syndrome coronavirus 2'OR 'severe acute respiratory syndrome cov 2' OR 'sars coronavirus 2'OR 'sars cov 2' OR ‘coronavirus’ ) AND AB (‘neuropsychology' OR 'neuropsychological' OR 'cognitive' OR 'cognition' OR 'brain' OR 'neurological' OR 'encephalitis')

**Table 1. Narrative description of included papers**

| Author(s) | Type | Short description | Methodology |
| --- | --- | --- | --- |
| Ahmad et al. 2020 (1) | Literature review | Next to the primary respiratory and cardiac manifestations neurological symptoms are also increasingly reported in case reports and case series, such as CVA, Guillain-Barré syndrome, acute transverse myelitis, and acute encephalitis. Most common peripheral manifestation was hyposmia. | Review of two articles |
| Al Saiegh et al. 2020 (2) | Case study | In two patients with a CVA, SARS-CoV-2 was not found in CSF. | Description two patients |
| Asadi-Pooya et al. 2020 (3) | Literature review | Neurological symptoms in COVID-19 are not well studied. However, it seems like the CNS is involved and this causes neurological symptoms. | Review eight studies |
| Asadi Pooya et al. 2020 (4) | Opinion paper | Both neurotropic and neuroinvasive capability of coronaviruses have been described. Although seizures do not often occur in COVID-19 patients it is important to treat seizures when it does. | N/A |
| Avula et al. 2020 (5) | Case study | Stroke in COVID-19 patients might be an atypical symptom. | Description four patients |
| Baig et al. 2020 (6) | Opinion paper | There are indications for the neuroinvasiveness of COVID-19. This might be related due to the presence of ACE2 in the brain. It is of interest to explore whether neurological damage contributes to morbidity and mortality in COVID-19. | N/A |
| Baig 2020 (7) | Letter to the editor | The loss of smell and taste can be an important symptom in COVID-19. The virus can reach the brain through the olfactory bulb or the blood after lung infection. | N/A |
| Baig 2020 (8) | Opinion paper | There is increasing evidence that the CNS is involved in COVID-19. Neurological symptoms are for example anosmia, dysgeusia, ataxia and changing consciousness. | N/A |
| Benny 2020 (9) | Opinion paper | Novel investigations show involvement of the central and peripheral nervous system in COVID-19 infection. However, neuromuscular manifestations are uncommon. | N/A |
| Bernard-Valnet et al. 2020 (10) | Case study | SARS-CoV-2 can present atypically. In two patients, acute meningoencephalitis is observed. | Description two patients |
| Betran-Recasens et al. 2020 (11) | Letter to the editor | The cytokine storm damages the neurons that regulate breathing. SARS-CoV-2 enters the CNS through the olfactory bulb. | N/A |
| Bostanciklioğl et al. 2020 (12) | Letter to the editor | The neuroinvasive character of SARS-CoV-2 can explain respiratory failure. The virus spreads through the olfactory bulb. Mechanisms for neurological symptoms are neuro-inflammation and thrombotic complications that can cause ischemic strokes. | N/A |
| Butowt et al. 2020 (13) | Opinion paper | ACE2 is present in the olfactory bulb which can cause loss of smell. This could also be the entrance to the brain and brain infections. When the virus is present in CSF it can reach the medulla oblongata that regulates breathing. | N/A |
| Calcagno et al. 2020 (14) | Letter to the editor | COVID-19 is primarily a respiratory diagnosis, but more evidence is emerging that points toward a neurologic component as well. It is likely neuroinvasion plays a role. | N/A |
| Carod-Artal 2020 (15) | Opinion paper | Other coronaviruses have already been shown to be neuroinvasive. Neurological symptoms are: headache, anosmia, acute necrotizing encephalopathy, stroke, brain seizures, and Guillain-Barré syndrome. | N/A |
| Chen et al. 2020 (16) | Research article | In 20% of COVID-19 patients brain damage was found due to hypoxic encephalopathy. | Observational study of deceased patients (n = 113) |
| Chigr et al. 2020 (17) | Reaction on Li et al. 2020 | SARS-CoV-2 influences the medulla oblongata which can cause respiratory failure | N/A |
| Condie 2020 (18) | Opinion paper | Neurotropic factors have been described by earlier studies in COVID-19, together with psychiatric comorbidity these patients may be extra vulnerable to a neural impact. Therefore, neuropsychological changes might be expected in children and adults that recovered from COVID-19. | N/A |
| Daou et al. 2020 (19) | Literature review | Earlier studies showed that coronaviruses are neuroinvasive and therefore it can be assumed that this is also the case in SARS-CoV-2. This deserves monitoring and timely recognition | Review 46 articles |
| Das et al. 2020 (20) | Opinion paper | Neurological symptoms can vary from mild to severe and implicate that the virus is neuroinvasive. | N/A |
| De Felice et al. 2020 (21) | Opinion paper | SARS-CoV-2 can directly lead to neurological changes, worsen premorbid neurological problems, and increase the vulnerability. Neuro-inflammation can trigger mechanisms that increase the incidence of neurodegenerative diseases. | N/A |
| De Santis 2020 (22) | Letter to the editor | SARS-CoV-2 causes neuro-inflammation. It enters the brain trough the olfactory bulb. | N/A |
| Dinkin et al. 2020 (23) | Case study | SARS-CoV-2 can present atypically. In two patients, double vision and weak eye muscles were observed. | Description two patients |
| Duong et al. 2020 (24) | Case study | SARS-CoV-2 can present atypically. In one patient, meningoencephalitis was observed. | Description one patient |
| Filatov et al. 2020 (25) | Case study | SARS-CoV-2 can present atypically. In one patient, encephalopathy was observed. | Description one patient |
| Finsterer et al. 2020 (26) | Letter to the editor | ACE2 receptors are also located in the nose and taste buds and are the most likely explanation of the loss of taste and smell in COVID-19. | N/A |
| Finsterer et al. 2020 (27) | Case study | There is increasing evidence that the CNS is involved resulting in neurological manifestations in COVID-19 patients. A case study was described about a COVID-19 patient with meningoencephalitis. | Description one patient |
| Gandhi et al. 2020 (28) | Opinion paper | SARS-CoV-2 can enter the brain via the olfactory bulb. From there it can reach other regions, such as the thalamus or the brainstem. It can infect the respiratory centers of the brain which causes respiratory failure. It is therefore important to screen patients for neurological symptoms. | N/A |
| Gautier et al. 2020 (29) | Letter to the editor | Loss of taste and smell can be a symptom in COVID-19. | N/A |
| Goldberg et al. 2020 (30) | Case study | SARS-CoV-2 can present atypically. In one patient, an acute ischemic stroke was described. | Description one patient |
| Guan et al. 2020 (31) | Letter to the editor | SARS-CoV-2 offers the possibility to gain more insight into the link between viral infections and risk of neurological diseases. | N/A |
| Gutiérrez-Ortiz et al. 2020 (32) | Case study | SARS-CoV-2 can present atypically. In one patient, the Miller Fisher syndrome was observed and in another patient Polyneuritis Cranialis. | Description two patients |
| Helms et al. 2020 (33) | Research article | In 58 patients 49 had neurological symptoms (84%), such as encephalopathy, confusion, agitation, and corticospinal symptoms. Two of the thirteen patients with an MRI scan showed signs of a stroke. | Observational study of ICU patients (n = 58) |
| Hess et al. 2020 (34) | Opinion paper | SARS-CoV-2 binds to ACE2 which causes a disbalance in the RAS-system (angiotensin I and II). This could be an explanation for the presentation of stroke in SARS-CoV-2. | N/A |
| Huang et al. 2020 (35) | Reaction on Duong et al. 2020 | Update on case report on COVID-19 patient with meningoencephalitis. CSF was found to be positive for SARS CoV-2. | Description one patient |
| Jiménez-Ruiz et al. 2020 (36) | Letter to the editor | There are neurological manifestations in COVID-19 patients, such as encephalitis, seizures, Guillain-Barré syndrome, disseminated encephalomyelitis and hemorrhagic leukoencephalitis. | N/A |
| Juliao Camaańo et al. 2020 (37) | Case study | SARS-CoV-2 can present atypically. In one COVID-19 patient, facial diplegia as possible atypical variant of Guillain-Barré syndrome is reported. | Description one patient |
| Kandemirli et al. 2020 (38) | Research paper | In 749 COVID-19 patients in 8 hospitals 235 were administered on an ICU (31%). Of these patients one out of five developed neurological symptoms (31%). Of the patients with an MRI scan 44% had abnormal findings. | Observational study in ICU patients with an MRI (n = 27) |
| Kaya et al. 2020 (39) | Letter to the editor | SARS-CoV-2 can present atypically. In one COVID-19 patient, transient cortical blindness in COVID-19 pneumonia was observed. | Description one patient |
| Klok et al. 2020 (40) | Research article | 31% of the 184 ICU patients with COVID-19 had thrombotic complications with ischemic stroke. | Observational study of ICU patients (n = 184) |
| Kuroda et al. 2020 (41) | Letter to the editor | Effect of COVID-19 in patients with epilepsy is unclear. | N/A |
| Li et al. 2020 (42) | Literature review | In some COVID-19 patients, neurological symptoms were observed (headache, nausea). In other coronaviruses it has been shown that they target the brainstem. This might be an explanation for respiratory failure in COVID-19. | Review of articles |
| Li et al. 2020 (43) | Opinion paper | End of February the first COVID-19 patients with neurological symptoms were described. It seems likely that the virus is neuroinvasive. | N/A |
| Li et al. 2020 (44) | Opinion paper | SARS-CoV-2 can have an effect on the brain via three pathways: hypoxia, neuro-inflammation, and ACE2. | N/A |
| Li et al. 2020 (45) | Literature review | COVID-19 is not limited to the respiratory system as neurological manifestations have also been reported. involvement of the CNS might indicate poor prognosis and worsening of disease. | Review of articles |
| Liguori et al. 2020 (46) | Research article | The majority (91.3%) of 103 patients with COVID-19 experienced subjective neurological symptoms of which sleep impairment was the most common. This was followed by dysgeusia, headache, hyposmia, and depression. Furthermore, women more frequently present subjective neurological symptoms than men. | Observational study of patients (n = 103) |
| Liu et al. 2020 (47) | Letter to the editor | Neurological symptoms can present in COVID-19 patients (e.g., headache, loss of taste and smell, stroke) | N/A |
| Lu et al. 2020 (48) | Research article | 27% of 304 patients with COVID-19 had brain seizures. | Observational multicenter study of patients (n = 304) |
| Mankad et al. (49) | Opinion paper | There are different pathways through which SARS-CoV-2 enters the brain (e.g., blood circulation, neuro-inflammation, olfactory bulb). | N/A |
| Mao et al. (50) | Research article | 36.4 COVID-19 patients had neurological symptoms which could me classified in four groups: acute cerebrovascular disease, impaired consciousness, peripheral nervous system involvement, and muscular manifestations. | Observational study of patients (n = 214) |
| Mao and Jin 2020 (51) | Opinion paper | Brain infection should be considered in COVID-19. Possible pathways might be: olfactory nerves, ACE2 and cytokine storms. A severe brain infection might be the cause of respiratory failure and high mortality rate. | N/A |
| Montalvan et al. 2020 (52) | Systematic review | SARS-CoV-2 is expressed in the nervous system. Common neurological symptoms are hyposmia, headaches, and altered consciousness. Associations have also been reported on encephalitis, demyelination, neuropathy, and stroke. One of the mechanisms proposed are invasion through the olfactory bulb. Invasion of SARS-CoV-2 of the medullary cardiorespiratory center might lead to respiratory failure. | Review of 67 papers |
| Moriguchi et al. (53) | Case study | SARS-CoV-2 can present atypically. In one patient encephalopathy was observed. | Description one patient |
| Muhammad et al. 2020 (54) | Case study | SARS-CoV-2 can present atypically. In one patient severe subarachnoid hemorrhage was observed. | Description one patient |
| Needham et al. 2020 (55) | Opinion paper | Possible mechanisms of neurological symptoms in COVID-19 are: - Non-immunological: hypotension, hypoxia, micro- and macrovascular thrombosis - Immunological: adaptive autoimmunity, activation microglia, maladaptive cytokine profile | N/A |
| Ng Kee Kwong et al. 2020 (56) | Literature review | The primary manifestation in COVID-19 is pneumonia. However, more evidence is being gathered for neurological manifestations, such as impaired consciousness, skeletal muscle injury, and acute cerebrovascular disease. Potential mechanisms might be the immune-related pathway via cytokine storms. | Review of articles |
| Paniz-Mondolfi et al. 2020 (57) | Case study | Post mortem evaluation showed the presence of SARS-CoV-2 in the frontal lobe | Description one deceased patient |
| Pereira 2020 (58) | Opinion paper | Neurological symptoms appear in COVID-19 patients and the virus most likely enters the brain via the olfactory bulb. | N/A |
| Poyiadji et al. 2020 (59) | Case study | SARS-CoV-2 can present atypically. In one patient acute necrotizing encephalopathy was observed. | Description one patient |
| Rodriguez-Morales et al. 2020 (60) | Opinion paper | Neurological symptoms are observed in COVID-1 patients (e.g., headache, ansomia, dysgeusia). | N/A |
| Roe 2020 (61) | Opinion paper | Reactivation and neurological symptoms in COVID-19 is in line with earlier research in RNA viruses. | N/A |
| Saveedra 2020 (62) | Opinion paper | SARS-CoV 2 is neuroinvasive and can damage the brain, this might be related to respiratory failure. | N/A |
| Scheidl et al. 2020 (63) | Case study | SARS-CoV-2 can present atypically. In one patien Guillan-Barré syndrome was observed. | Description one patient |
| Sellner et al. 2020 (64) | Letter to the editor | Due to the presence of neurological symptoms in COVID-19 it is important that a neurologist is involved in the multidisciplinary care. Furthermore, the neuroinvasive role might explain respiratory failure. | N/A |
| Sepehrinezhad et al. 2020 (65) | Literature review | Other coronaviruses have neuroinvasive properties, likely via olfactory epithelium, cellular infection, blood brain barrier, and trans-synaptic transmission. Neurological symptoms in COVID-19 are headaches, nausea, confusion, dizziness, impaired consciousness, ataxia, acute cerebrovascular diseases, vomiting, epilepsy, and skeletal muscle symptoms. | Review of articles |
| Serrano-Castro et al. 2020 (66) | Literature review | SARS-CoV-2 is a neuroinvasive virus which can cause a cytokine storm and a neuroinflammatory response. | Review of articles |
| Shaikh et al. 2020 (67) | Opinion paper | Neurological manifestation in the early phase of SARS-CoV-2 are: confusion, dizziness, stroke, anosmia, dysgeusia, ataxia, epilepsy, and nerve pain. | N/A |
| Singh et al. 2020 (68) | Opinion paper | It is still unclear whether SARS-CoV-2 directly affects nerve systems in humans. The authors hypothesize that most neurological symptoms are non-specific and secondary to the illness. | N/A |
| Sohal et al. 2020 (69) | Case study | SARS-CoV-2 can present atypically. In one patient multiple brain seizures were observed. | Description one patient |
| Soman et al. 2020 (70) | Case study | SARS-CoV-2 can present atypically. In one patient de novo status epilepticus was observed and in the other patient pneumonia and fluctuating mental status that led to suspicion of status epilepticus. | Description two patients |
| Steardo et al. 2020 (71) | Opinion paper | Neurological symtpoms are observed in SARS-CoV-2. Furthermore, neuroinvasiveness is suspected in the brainstem. Tis could explain respiratory failure. Neuro-inflammation is caused by the cytokine storm via the blood brain barrier. | N/A |
| Tassorelli et al. 2020 (72) | Letter to the editor | Respiratory failure in SARS-CoV-2 can be explained due to dysfunction of the respiratory centers in the brainstem. | N/A |
| Toljan 2020 (73) | Reaction on Baig 2020 | SARS-CoV-2 is neuroinvasive due to the presence of ACE2 in the brain, via the lymphatic system and via neuro-inflammation. | N/A |
| Vonck et al. 2020 (74) | Literature review | Neurological manifestations have been reported in COVID-19 patients. This might be the result of dysregulation of homeostasis. | Review of 20 articles |
| Wang et al. 2020 (75) | Letter to the editor | The following neurological symptoms are observed in COVID-19 patients: headache, instable walking, and stroke. | N/A |
| Werner et al. 2020 (76) | Literature review | Neurological manifestations vary from mild to severe (e.g. encephalitis. It is unclear whether these are due to direct viral injury or due to the systemic disease. The virus may be neuroinvasive. | Review of multiple studies (e.g. 6 case reports) |
| Whittaker et al. 2020 (77) | Literature review | Headache and anosmia are common neurological symptoms in COVID-19 patients. Less common are seizure, stroke, and Guillain-Barré syndrome. | Review of 31 articles |
| Wilson et al. 2020 (78) | Scoping review | COVID-19 may manifest as neurological symptoms, including headache, dizziness, hypogeusia, anosmia, altered level of consciousness, acute cerebrovascular events, seizure and ataxia. COVID-19 appears to have a neuroinvasive potential. Long-term effects on neurodegeneration still have to be cleared up. | Review of 29 articles |
| Wu et al. 2020 (79) | Opinion paper | Other coronaviruses have been found in CSF. Possible mechanisms of neurological damage is: direct infection pathways (blood circulation, neuronal pathways), hypoxia, ACE2, immune disorders. SARS-CoV-2 can enter the brain via the olfactory bulb and blood circulation. | N/A |
| Ye et al. 2020 (80) | Case study | SARS-CoV-2 can present atypically. In one patient encephalopathy was observed. | Description one patient |
| Yin et al. 2020 (81) | Case study | SARS-CoV-2 can present atypically. In one patient altered consciousness and psychiatric symptoms were observed. | Description one patient |
| Zanin et al. 2020 (82) | Case study | SARS-CoV-2 can present atypically. Headache, anosmia, and dysgeusia are common symptoms. Both altered consciousness and brain seizures can also occur. | Description one patient |
| Zhao et al. 2020 (83) | Case study | SARS-CoV-2 can present atypically. In one patient Guillain-Barré syndrome was observed | Description one patient |
| Zhou et al. 2020 (84) | Opinion paper | Neurological symptoms are reported in SARS-CoV-2. | N/A |
| Zhou et al. 2020 (85) | Letter to the editor | The question is raised whether of SARS-CoV-2 can cause lasting brain damage which might lead to the development of neurological diseases. | N/A |

**References**

1. Ahmad I, Rathore FA. Neurological manifestations and complications of COVID-19: A literature review. Preprints. 2020. doi: 10.20944/preprints202004.0453.v1

2. Al Saiegh F, Ghosh R, Leibold A, Avery MB, Schmidt RF, Theofanis T, et al. Status of
 SARS-CoV-2 in cerebrospinal fluid of patients with COVID-19 and stroke. J Neurol
 Neurosurg Psychiatry. 2020. doi: 10.1136/jnnp-2020-323522

3. Asadi-Pooya AA, Simani L. Central nervous system manifestations of COVID-19: A
 systematic review. J Neurol Sci. 2020;413:116832.

4. Asadi-Pooya AA. Seizures associated with coronavirus infections. Seizure. 2020;79:49-52.

5. Avula A, Nalleballe K, Narula N, Sapozhnikov S, Dandu V, Toom S, et al. COVID-19 presenting as stroke. Brain Behav Immun. 2020. doi: [10.1016/j.bbi.2020.04.077](https://dx.doi.org/10.1016%2Fj.bbi.2020.04.077)

6. Baig AM, Khaleeq A, Ali U, Syeda H. Evidence of the COVID-19 virus targeting the CNS: Tissue distribution, host-virus interaction, and proposed neurotropic mechanisms. ACS Chem Neurosci. 2020;11(7):995-8.

7. Baig AM. Neurological manifestations in COVID-19 caused by SARS-CoV-2. CNS Neurosci
 Ther. 2020;26(5):499-501.

8. Baig AM. Updates on what ACS Reported: Emerging evidences of COVID-19 with nervous system involvement. ACS Chem Neurosci. 2020. doi: 10.1021/acschemneuro.0c00181

9. Benny R, Khadilkar SV. COVID 19: Neuromuscular manifestations. Ann Indian Acad Neurol. 2020;23(Suppl 1):S40-s2.

10. Bernard-Valnet R, Pizzarotti B, Anichini A, Demars Y, Russo E, Schmidhauser M, et al. Two
 patients with acute meningo-encephalitis concomitant to SARS-CoV-2 infection. Eur J
 Neurol. 2020. doi: 10.1111/ene.14298

11. Bertran Recasens B, Martinez-Llorens JM, Rodriguez-Sevilla JJ, Rubio MA. Lack of dyspnea
 in COVID-19 patients; another neurological conundrum? Eur J Neurol. 2020. doi:
 10.1111/ene.14265.

12. Bostanciklioğlu M. SARS-CoV2 entry and spread in the lymphatic drainage system of the
 brain. Brain Behav Immun. 2020. doi: [10.1016/j.bbi.2020.04.080](https://dx.doi.org/10.1016%2Fj.bbi.2020.04.080)

13. Butowt R, Bilinska K. SARS-CoV-2: Olfaction, brain Infection, and the urgent need for
 clinical samples allowing earlier virus detection. ACS Chem Neurosci. 2020. doi:
 10.1021/acschemneuro.0c0017

14. Calcagno N, Colombo E, Maranzano A, Pasquini J, Keller Sarmiento IJ, Trogu F, et al. Rising evidence for neurological involvement in COVID-19 pandemic. Neurol Sci. 2020:1-3. doi: 10.1007/s10072-020-04447-w

15. Carod-Artal FJ. Neurological complications of coronavirus and COVID-19. Rev Neurologia.
 2020;70(9):311-22.

16. Chen T, Wu D, Chen H, Yan W, Yang D, Chen G, et al. Clinical characteristics of 113
 deceased patients with coronavirus disease 2019: retrospective study. BMJ. 2020;368:m1091.

17. Chigr F, Merzouki M, Najimi M. Comment on "The neuroinvasive potential of SARS-CoV2
 may play a role in the respiratory failure of COVID-19 patients". J Med Virol. 2020. doi:
 10.1002/jmv.25960

18. Condie LO. Neurotropic mechanisms in COVID-19 and their potential influence on neuropsychological outcomes in children. Child Neuropsychol. 2020:1-20.doi: 10.1080/09297049.2020.1763938

19. Daou BJ, Koduri S, Palmateer G, Thompson BG, Chaudhary N, Gemmete JJ, et al. Letter: Neurological implications of COVID-19 and lessons learned from prior epidemics and pandemics. Neurosurgery. 2020. doi: 10.1093/neuros/nyaa186

20. Das G, Mukherjee N, Ghosh S. Neurological insights of COVID-19 pandemic. ACS Chem Neurosci. 2020;11(9):1206-9.

21. De Felice FG, Tovar-Moll F, Moll J, Munoz DP, Ferreira ST. Severe acute respiratory
 syndrome Coronavirus 2 (SARS-CoV-2) and the central nervous system. Trends Neurosci.
 2020. doi: 0.1016/j.tins.2020.04.004

22. De Santis G. SARS-CoV-2: A new virus but a familiar inflammation brain pattern. Brain
 Behav Immun. 2020. doi: 10.1016/j.bbi.2020.04.066

23. Dinkin M, Gao V, Kahan J, Bobker S, Simonetto M, Wechsler P, et al. COVID-19 presenting with ophthalmoparesis from cranial nerve palsy. Neurology. 2020. doi: 10.1212/WNL.0000000000009700

24. Duong L, Xu P, Liu A. Meningoencephalitis without respiratory failure in a young female patient with COVID-19 infection in downtown Los Angeles, early April 2020. Brain Behav Immun. 2020. doi: 10.1016/j.bbi.2020.04.024

25. Filatov A, Sharma P, Hindi F, Espinosa PS. Neurological complications of coronavirus disease (COVID-19): Encephalopathy. Cureus. 2020; 12(30:e7352.

26. Finsterer J, Stollberger C. Causes of hypogeusia/hyposmia in SARS-CoV2 infected patients. J
 Med Virol. 2020. doi: 10.1002/jmv.25903

27. Finsterer J, Stollberger C. Update on the neurology of COVID-19. J Med Virol. 2020. doi: 1[0.1002/jmv.26000](https://doi.org/10.1002/jmv.26000)

28. Gandhi S, Srivastava AK, Ray U, Tripathi PP. Is the collapse of the respiratory center in the brain Responsible for respiratory breakdown in COVID-19 patients? ACS Chem Neurosci. 2020. doi: 10.1021/acschemneuro.0c00217

29. Gautier J-F, Ravussin Y. A New Symptom of COVID-19: Loss of taste and smell. Obesity.
 2020;28(5):848.

30. Goldberg MF, Goldberg MF, Cerejo R, Tayal AH. Cerebrovascular Disease in COVID-19. AJNR Am J Neuroradiol. 2020. doi: 10.3174/ajnr.A6588

31. Guan W-j, Ni Z-y, Hu Y, Liang W-h, Ou C-q, He J-x, et al. Clinical Characteristics of Coronavirus Disease 2019 in China. N Engl J Med. 2020. doi: 10.1056/NEJMoa2002032

32. Gutiérrez-Ortiz C, Méndez A, Rodrigo-Rey S, San Pedro-Murillo E, Bermejo-Guerrero L,
 Gordo-Mañas R, et al. Miller Fisher syndrome and polyneuritis cranialis in COVID-19.
 Neurology. 2020. doi: 10.1212/WNL.0000000000009619

33. Helms J, Kremer S, Merdji H, Clere-Jehl R, Schenck M, Kummerlen C, et al. Neurologic
 features in severe SARS-CoV-2 infection. N Engl J Med. 2020. doi: 10.1056/NEJMc2008597

34. Hess DC, Eldahshan W, Rutkowski E. COVID-19-related stroke. Transl Stroke Res. 2020;
 11:322-5.

35. Huang YH, Jiang D, Huang JT. A case of COVID-19 encephalitis Brain Behav Immun. 2020.
 doi: [10.1016/j.bbi.2020.05.012](https://dx.doi.org/10.1016%2Fj.bbi.2020.05.012)

36. Jiménez-Ruiz A, García-Grimshaw M, Ruiz-Sandoval JL. Neurological manifestations of COVID-19. Gac Med Mex. 2020;156(4).

37. Juliao Caamaño DS, Alonso Beato R. Facial diplegia, a possible atypical variant of Guillain-Barré Syndrome as a rare neurological complication of SARS-CoV-2. J Clin Neurosci. 2020. doi: 10.1016/j.jocn.2020.05.016

38. Kandemirli SG, Dogan L, Sarikaya ZT, Kara S, Akinci C, Kaya D, et al. Brain MRI findings
 in patients in the intensive care unit with COVID-19 infection. Radiology. 2020:201697.

39. Kaya Y, Kara S, Akinci C, Kocaman AS. Transient cortical blindness in COVID-19 pneumonia; a PRES-like syndrome: Case report. J Neurol Sci. 2020;413:116858.

40. Klok FA, Kruip MJHA, van der Meer NJM, Arbous MS, Gommers DAMPJ, Kant KM, et al.
 Incidence of thrombotic complications in critically ill ICU patients with COVID-19.
 Thromb Res. 2020. doi: 10.1016/j.thromres.2020.04.013

41. Kuroda N. Epilepsy and COVID-19: Associations and important considerations. Epilepsy Behav. 2020;108:107122.

42. Li YC, Bai WZ, Hashikawa T. The neuroinvasive potential of SARS-CoV2 may play a role in the respiratory failure of COVID-19 patients. J Med Virol. 2020. doi: 10.1002/jmv.25728

43. Li Z, Huang Y, Guo X. The brain, another potential target organ, needs early protection from
 SARS-CoV-2 neuroinvasion. Sci China Life Sci. 2020;63(5):771-3.

44. Li Z, Liu T, Yang N, Han D, Mi X, Li Y, et al. Neurological manifestations of patients with
 COVID-19: potential routes of SARS-CoV-2 neuroinvasion from the periphery to the brain.
 Front Med. 2020. doi: 10.1007/s11684-020-0786-5

45. Li H, Xue Q, Xu X. Involvement of the nervous system in SARS-CoV-2 infection. Neurotox Res. 2020;38(1):1-7.

46. Liguori C, Pierantozzi M, Spanetta M, Sarmati L, Cesta N, Iannetta M, et al. Subjective
 neurological symptoms frequently occur in patients with SARS-CoV2 infection. Brain Behav
 Immun. 2020. doi: 10.1016/j.bbi.2020.05.037

47. Liu K, Pan M, Xiao Z, Xu X. Neurological manifestations of the coronavirus (SARS-CoV-2)
 pandemic 2019-2020. J Neurol Neurosurg Psychiatry. 2020. doi: [10.3389/fneur.2020.00498](https://doi.org/10.3389/fneur.2020.00498)

48. Lu L, Xiong W, Liu D, Liu J, Yang D, Li N, et al. New onset acute symptomatic seizure and
 risk factors in coronavirus disease 2019: A retrospective multicenter study. Epilepsia. 2020.
 doi: 10.1111/epi.16524.

49. Mankad K, Perry MD, Mirsky DM, Rossi A. COVID-19: A primer for nuroradiologists.
 Neuroradiology. 2020. doi: [10.1007/s00234-020-02437-5](https://dx.doi.org/10.1007%2Fs00234-020-02437-5) 

50. Mao L, Wang M, Chen S, He Q, Chang J, Hong C, et al. Neurological manifestations of
 hospitalized patients with COVID-19 in Wuhan, China: a retrospective case series study. J
 AMA Neurol. 2020. doi:10.1001/jamaneurol.2020.1127.

51. Mao XY, Jin WL. The COVID-19 pandemic: Consideration for brain infection. Neuroscience. 2020;437:130-1.

52. Montalvan V, Lee J, Bueso T, De Toledo J, Rivas K. Neurological manifestations of COVID-19 and other coronavirus infections: A systematic review. Clin Neurol Neurosurg. 2020;194:105921.

53. Moriguchi T, Harii N, Goto J, Harada D, Sugawara H, Takamino J, et al. A first case of meningitis/encephalitis associated with SARS-Coronavirus-2. Int J Infect Dis. 2020;94:55-8.

54. Muhammad S, Petridis A, Cornelius JF, Hänggi D. Letter to editor: Severe brain haemorrhage and concomitant COVID-19 Infection: A neurovascular complication of COVID-19. Brain Behav Immun. 2020. doi: [10.1016/j.bbi.2020.05.015](https://dx.doi.org/10.1016%2Fj.bbi.2020.05.015)

55. Needham EJ, Chou SH, Coles AJ, Menon DK. Neurological implications of COVID-19
 infections. Neurocrit Care. 2020:1-5. doi: 10.1007/s12028-020-00978-4

56. Ng Kee Kwong KC, Mehta PR, Shukla G, Mehta AR. COVID-19, SARS and MERS: A neurological perspective. J Clin Neurosci. 2020. doi: 10.1016/j.jocn.2020.04.124

57. Paniz-Mondolfi A, Bryce C, Grimes Z, Gordon RE, Reidy J, Lednicky J, et al. Central
 nervous system involvement by severe acute respiratory syndrome coronavirus - 2 (SARS-
 CoV-2). J Med Virol. 2020. doi: 10.1002/jmv.25915

58. Pereira A. Long-term neurological threats of COVID-19: A call to update the thinking about the outcomes of the coronavirus pandemic. Front Neurol. 2020;11:308.

59. Poyiadji N, Shahin G, Noujaim D, Stone M, Patel S, Griffith B. COVID-19–associated acute
 hemorrhagic necrotizing encephalopathy: CT and MRI Features. Radiology. 2020:201187.

60. Rodriguez-Morales AJ, Rodriguez-Morales AG, Méndez CA, Hernández-Botero S. Tracing new clinical manifestations in patients with COVID-19 in Chile and its potential relationship with the SARS-CoV-2 divergence. Curr Trop Med Rep. 2020:1-4. doi: [10.1007/s40475-020-00205-2](https://dx.doi.org/10.1007%2Fs40475-020-00205-2)

61. Roe K. Explanation for COVID-19 infection neurological damage and reactivations. Transbound Emerg Dis. 2020. doi: 10.1111/tbed.13594

62. Saavedra JM. COVID-19, Angiotensin Receptor Blockers, and the Brain. Cell Mol Neurobiol. 2020. doi: 10.1007/s10571-020-00861-y

63. Scheidl E, Canseco DD, Hadji-Naumov A, Bereznai B. Guillain-Barre syndrome during
 SARS-CoV-2 pandemic: a case report and review of recent literature. J Peripher Nerv Syst.
 2020. doi: 10.1111/jns.12382

64. Sellner J, Taba P, Öztürk S, Helbok R. The need for neurologists in the care of COVID-19 patients. Eur J Neurol. 2020. doi: 10.1111/ene.14257

65. Sepehrinezhad A, Shahbazi A, Negah SS. COVID-19 virus may have neuroinvasive potential
 and cause neurological complications: a perspective review. J Neurovirol. 2020. doi:
 [10.1007/s13365-020-00851-2](https://dx.doi.org/10.1007%2Fs13365-020-00851-2) 

66. Serrano-Castro PJ, Estivill-Torrús G, Cabezudo-García P, Reyes-Bueno JA, Ciano Petersen N, Aguilar-Castillo MJ, et al. Impact of SARS-CoV-2 infection on neurodegenerative and neuropsychiatric diseases: a delayed pandemic? Neurologia. 2020. doi: 10.1016/j.nrl.2020.04.002

67. Shaikh AG, Mitoma H, Manto M. Cerebellar scholars' challenging time in COVID-19 pandemia. Cerebellum. 2020;19(3):343-4.

68. Singh AK, Bhushan B, Maurya A, Mishra G, Singh SK, Awasthi R. Novel coronavirus d isease 2019 (COVID-19) and neurodegenerative disorders. Dermatol Ther. 2020:e13591.

69. Sohal S, Mossammat M. COVID-19 Presenting with seizures. IDCases. 2020:e00782.

70. Somani S, Pati S, Gaston T, Chitlangia A, Agnihotri S. De novo status epilepticus in patients with COVID-19. Ann Clin Transl Neurol. 2020. doi: 10.1002/acn3.51071

71. Steardo L, Steardo L, Jr., Zorec R, Verkhratsky A. Neuroinfection may contribute to
 pathophysiology and clinical manifestations of COVID-19. Acta Physiol (Oxf). 2020:e13473.
 doi: 10.1111/apha.13473

72. Tassorelli C, Mojoli F, Baldanti F, Bruno R, Benazzo M. COVID-19: what if the brain had a role in causing the deaths? Eur J Neurol. 2020. doi: 10.1111/ene.14275

73. Toljan K. Letter to the Editor Regarding the Viewpoint "Evidence of the COVID-19 virus
 targeting the CNS: tissue distribution, host-virus Interaction, and proposed neurotropic
 mechanism". ACS Chem Neurosci. 2020;11(8):1192-4

74. Vonck K, Garrez I, De Herdt V, Hemelsoet D, Laureys G, Raedt R, et al. Neurological manifestations and neuro-invasive mechanisms of the severe acute respiratory syndrome coronavirus type 2. Eur J Neurol. 2020. doi: 10.1111/ene.14329

75. Wang H, Li X-L, Yan Z-R, Sun X-P, Han J, Zhang B-W. Potential neurological symptoms of COVID-19. Ther Adv Neurol Disord. 2020;13:1756286420917830.

76. Werner C, Scullen T, Mathkour M, Zeoli T, Beighley A, Kilgore MD, et al. Neurological
 impact of coronavirus disease (COVID-19): Practical considerations for the neuroscience
 community. World Neurosurg. 2020. doi: 10.1016/j.wneu.2020.04.222

77. Whittaker A, Anson M, Harky A. Neurological Manifestations of COVID-19: A review. Acta Neurol Scand. 2020. doi: 10.1111/ane.13266

78. Wilson MP, Jack AS. Coronavirus disease 2019 (COVID-19) in neurology and neurosurgery: A scoping review of the early literature. Clin Neurol Neurosurg. 2020;193:105866.

79. Wu Y, Xu X, Chen Z, Duan J, Hashimoto K, Yang L, et al. Nervous system involvement after
 infection with COVID-19 and other coronaviruses. Brain Behav Immun. 2020. doi:
 10.1016/j.bbi.2020.03.031

80. Ye M, Ren Y, Lv T. Encephalitis as a clinical manifestation of COVID-19. Brain Behav Immun. 2020. doi: [10.1016/j.bbi.2020.04.017](https://dx.doi.org/10.1016%2Fj.bbi.2020.04.017)

81. Yin R, Feng W, Wang T, Chen G, Wu T, Chen D, et al. Concomitant neurological symptoms observed in a patient diagnosed with coronavirus disease 2019. J Med Virol. 2020. doi: 10.1002/jmv.25888

82. Zanin L, Saraceno G, Panciani PP, Renisi G, Signorini L, Migliorati K, et al. SARS-CoV-2 can induce brain and spine demyelinating lesions. Acta Neurochir (Wien). 2020. doi: [0.1007/s00701-020-04374-x](https://dx.doi.org/10.1007%2Fs00701-020-04374-x)

83. Zhao J, Rudd A, Liu R. Challenges and potential solutions of stroke care during the coronavirus disease 2019 (COVID-19) outbreak. Stroke. 2020;51(5):1356-7.

84. Zhou Y, Li W, Wang D, Mao L, Jin H, Li Y, et al. Clinical time course of COVID-19, its neurological manifestation and some thoughts on its management. Stroke Vasc Neurol. 2020. doi: 10.1136/svn-2020-000398

85. Zhou L, Zhang M, Wang J, Gao J. Sars-Cov-2: Underestimated damage to nervous system. Travel Med Infect Di. 2020:101642.
